# A Flexible Wearable Electronics System for Electrocardiographic Assessment of Colchicine Therapy for Post-MI Remodeling

**DOI:** 10.3390/s26092814

**Published:** 2026-04-30

**Authors:** Weijia Huang, Xiangfeng Gong, Maoshuai Yang, Ting Huang, Qiyao Zhuang, Zhenghua Xiao, Tao Xiong, Gang Yang

**Affiliations:** 1 College of Biomedical Engineering, Sichuan University, Chengdu 610065, China; 2 Department of Cardiovascular Surgery, West China Hospital of Sichuan University, Chengdu 610041, China; 3 Department of Pediatrics, West China Second University Hospital of Sichuan University, Chengdu 610041, China

**Keywords:** flexible electronics, wearable devices, electrocardiogram, myocardial infarction, cardiac remodeling

## Abstract

**Objective:** Myocardial infarction (MI) triggers inflammation and fibrosis that drive the progressive impairment of cardiac function. Yet most pharmacological studies still depend on single-time-point histological or imaging endpoints and lack longitudinal, non-invasive assessments of treatment response. Electrocardiography (ECG) detects conduction and repolarization abnormalities tightly associated with myocardial injury and structural remodeling. However, ECG monitoring in mice is limited by rigid or invasive hardware, which restricts its use for longitudinal assessment of cardiac structure and function. **Approach:** Here, we propose an ECG-based non-invasive post-MI cardiac remodeling assessment approach and develop a flexible electrocardiographic monitoring microsystem (FECMS). Using the anti-remodeling drug (colchicine) therapy in an MI mouse model (Sham *n* = 4, MI *n* = 7 survivors, Col *n* = 7 survivors) for validation, we longitudinally track drug-induced changes in ECG parameters and systematically evaluate their concordance with functional, structural, and molecular indicators of cardiac injury and remodeling. **Results:** Colchicine treatment induced progressive shortening of the QRS and QT intervals and gradual stabilization of the PR interval. These interval changes were accompanied by increased EF and FS, decreased LVESV, reduced myocardial fibrosis and inflammatory infiltration, and lower plasma troponin I levels at the endpoint. Correlation analyses revealed strong relationships between drug-induced changes in ECG parameters and functional recovery and inhibited structural remodeling. **Significance:** The FECMS provides a new, non-invasive tool for longitudinal cardiovascular drug evaluation. This approach has the potential to complement or reduce reliance on terminal histological endpoints and to facilitate the optimization of dosing strategies in preclinical cardiovascular pharmacology.

## 1. Introduction

Myocardial infarction (MI) remains a leading cause of mortality worldwide [1]. The inflammation triggered by MI promotes cardiomyocyte death and drives the gradual decline of cardiac function and structural remodeling, which can ultimately culminate in heart failure [2]. Furthermore, because post-MI intervention and its effects unfold over time, treatment efficacy cannot be inferred from endpoint measurements alone [3,4,5]. Thus, post-MI remodeling and intervention research is intrinsically longitudinal, with complementary longitudinal readouts needed to capture temporal changes in remodeling and treatment response and to support the translation of fundamental findings into clinically relevant insights.

Traditional evaluation of cardiac injury and remodeling in MI mice relies on imaging and terminal assessments. Non-invasive tools such as echocardiography and cardiac magnetic resonance (CMR) assess cardiac function under controlled anesthesia. Invasive approaches, including histological staining and plasma biomarkers (such as N-terminal pro-B-type natriuretic peptide, soluble suppression of tumorigenicity 2 and extracellular matrix proteins), provide information at the tissue and molecular levels for MI model establishment and drug evaluation. These approaches are indispensable and provide strong biological interpretability. But they also have limitations that cannot be overlooked, including costly equipment and reagents, dependence on image quality and operator expertise, high sensitivity to cardiac size and heart rate in small animals, and an inability to achieve continuous monitoring. Consequently, there is a pressing need for new methods that can provide non-invasive, dynamic, and longitudinal monitoring of the course of cardiac remodeling and functional changes in the field of cardiovascular drug development.

Electrocardiography (ECG), which provides direct signal of cardiac conduction and repolarization, has been widely applied in MI and ischemia–reperfusion models [6,7,8,9]. Animal research has shown that prolongation of ECG intervals (such as QRS and QT intervals) is frequently observed, which is associated with infarct scarring, inflammation, reduced ejection fraction and ventricular dilatation [10,11]. Recent clinical research further supports the relevance of ECG intervals to prognosis and remodeling. In acute MI, QT interval prolongation is associated with higher mortality and malignant ventricular arrhythmias [12,13]. In structural heart disease, prolonged QRS is associated with greater ventricular scar burden and adverse remodeling [14]. These findings suggest that ECG intervals have potential value as indicators of electrophysiological abnormalities and structural remodeling. However, most existing ECG studies have focused on disease diagnosis or risk stratification, with their use as serial treatment-responsive readouts in post-MI models, particularly during pharmacological intervention, remaining insufficiently explored.

Systematically and quantitatively using ECG intervals to track post-MI remodeling remains limited, largely because existing ECG monitoring systems suffer from technical constraints. Implantable ECG systems require technically demanding, highly invasive procedures, entail prolonged recovery and high costs, carry risks of postoperative complications and are poorly suited to large-scale experimental studies [11,15,16]. Rigid devices for surface ECG are bulky and hard, restrict movement and trigger stress, while device-related changes in ECG signals are difficult to distinguish from cardiac remodeling, which limits their suitability for long-term ECG monitoring [17,18]. More recent wearable or integrated monitoring systems have further advanced less invasive ECG acquisition, but there remains room to improve flexibility, weight, and longitudinal applicability in disease-treatment settings [19]. Together, these prior studies establish the feasibility of non-invasive or repeated ECG recording, while also indicating the need for flexible and lightweight implementations better adapted for small-animal use and for evaluating whether serial ECG interval changes may provide complementary readouts of treatment response after MI.

In this study, we investigated whether serial ECG intervals could provide a useful longitudinal readout of treatment-associated changes after MI. To enable stable, low-noise, multi-time-point ECG acquisition in mice, we designed and developed a flexible electrocardiographic monitoring microsystem (FECMS). We examined ECG-based assessment in a mouse model of MI under anti-remodeling drug treatment, using colchicine to generate different degrees of cardiac remodeling within the MI model. By combining multi-time-point ECG monitoring with functional, structural, and molecular analyses, we systematically evaluated whether longitudinal changes in ECG intervals tracked remodeling changes associated with treatment response after MI. This study aimed to explore the feasibility of using serial ECG intervals obtained from FECMS as a complementary approach for longitudinal assessment of post-MI remodeling and treatment response in a colchicine-treated mouse model.

## 2. Materials and Methods

### 2.1. Design and Implementation of the FECMS

**Figure 1:**
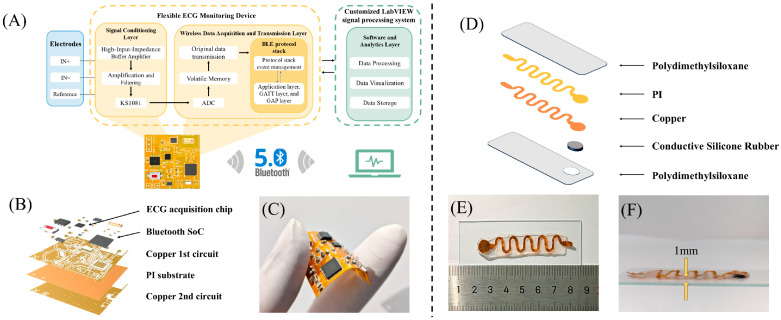
Structural design and prototype of the FECMS hardware and the flexible serpentine electrode. (**A**) System block diagram. (**B**) Hardware architecture schematic. (**C**) Demonstration of hardware flexibility. (**D**) Schematic illustration of the electrode structure. (**E**) Physical display of the electrode. (**F**) Side view of the electrode.

To achieve ECG-based dynamic assessment of cardiac remodeling and support multi-time-point follow-up of small animal models, this study constructed the FECMS. Representative ECG monitoring approaches and the positioning of the present FECMS are summarized in Supplementary Table S1. The system architecture is illustrated in Figure 1A. The system consists of wearable acquisition nodes and custom software. The acquisition nodes are responsible for ECG signal collection, data digitization, and wireless transmission, while the custom software handles data storage, signal processing, and real-time display. The acquisition node is constructed on a flexible polyimide (PI) substrate in the form of a flexible printed circuit board (FPCB). It integrates an ECG acquisition front-end (KS1081, Kingsense Electronics, Shenzhen, China) with high input impedance, integrated amplification and prefiltering, a low-power Bluetooth chip (nRF52832, Nordic Semiconductor, Trondheim, Norway), and a voltage-regulated power management module (LP3990-3.3, Texas Instruments, Dallas, TX, USA), all powered by a lithium battery. Timer-based sampling control was implemented using the high-precision internal MCU clock, with an ECG acquisition period of 500 Hz. During peripheral initialization, the ADC was configured in single-conversion mode with 12-bit resolution. The structural configuration of the acquisition node is shown in Figure 1B, and Figure 1C illustrates the device under flexion. The custom software was developed in LabVIEW 2020 (National Instruments, Austin, TX, USA) on a computer platform for real-time visualization of the acquired signals. Signal preprocessing in LabVIEW was used to reduce baseline drift and broadband noise, with baseline wander removed by the WADetrend VI (0.5 Hz cutoff) and broadband noise suppressed by the Wavelet Denoise Express VI using soft-thresholding, discrete wavelet transform, and the minimax rule. The wearable acquisition node weighing only 0.78 g and the 29 × 29 × 0.5 mm module is sufficiently compact and lightweight for portable ECG monitoring in mice. The system operated at an average current of approximately 11 mA, with a 100 mAh lithium battery providing a continuous runtime of about 9 h. Wireless transmission remained stable during operation, with a packet loss rate below 0.25%. In this study, we designed a polyester fiber vest to accommodate the acquisition node in a pocket located on the back of the wearable structure. The flexible serpentine electrode is routed through holes in the vest and attached to the mice’s body surface. Before the formal experiment, the mice underwent adaptation training to wear the vest and reduce stress responses.

### 2.2. Design and Fabrication of Flexible Serpentine Electrodes

To achieve a skin electrode combining stretchability, low impedance and high signal stability, we designed and fabricated a flexible serpentine electrode composed of a conductive layer, structural interconnects and an insulating encapsulation layer, as shown in Figure 1D. First, the conductive layer was prepared by mixing conductive silicone rubber (BD-7160, Zhejiang Powerwell Advanced Material Technology, Jinhua, China) with a catalyst at a 10:1 ratio. The mixture was coated onto a glass substrate. Serpentine copper traces (0.11 mm thick; rounded corners; 4 mm amplitude and spacing) were cut from copper foil using a cutting machine (SILHOUETTE CAMEO^®^ 4, Silhouette, Lindon, UT, USA), reinforced on the reverse side with a polyimide (PI) backing layer, and then placed onto the uncured conductive mixture. The serpentine design and PI reinforcement were utilized to distribute strain and maintain stable skin–electrode contact during the movement of the mice. The conductive layer was formed by curing on a thermostatic platform (X2026, Guangdong Xinhao Mai, Shenzhen, China) at 80 °C for 10 min. For the insulating encapsulation layer, polydimethylsiloxane (PDMS, SYLGARD 184) was mixed at a 28:1 (base: curing agent) ratio, degassed for 30 min to eliminate air bubbles, and poured over the glass substrate. Following a final curing step at 80 °C for 30 min, the completed electrodes were carefully peeled from the glass slide using a blade. As shown in Figure 1E,F, the resulting electrode is ultralight (total mass < 0.7 g, 1 mm thick), ultrathin and highly adherent, maintaining stable contact impedance and signal consistency during measurement, thereby providing a reliable ECG input to the FECMS.

### 2.3. Animal Model and Drug Intervention

#### 2.3.1. Mouse Model

This study used 20 C57 mice (male, 6–8 weeks of age) randomized into sham-operated group (Sham, *n* = 4, with 4 animals surviving to the endpoint), a myocardial infarction group (MI, *n* = 8; with 7 animals surviving to the endpoint), and a colchicine-treated group (Col, *n* = 8, with 7 animals surviving to the endpoint). Animals were housed under controlled environmental conditions (21–22 °C; 40–60% humidity; 12 h light/dark; ad libitum food and water). All animal experiments were conducted under a project license (20240112005) granted by the Animal Ethics Committee of West China Hospital, Sichuan University, in accordance with its guidelines for the care and use of laboratory animals. A summary of animal numbers at baseline, post-surgery, and study end point is provided in Supplementary Table S2.

#### 2.3.2. Induction of MI

Mice were anaesthetized using an inhalation anesthesia system, with 3% isoflurane in oxygen for induction for 90 s, followed by 2% isoflurane for maintenance. After disinfection of the surgical field with 75% ethanol, a 12 mm longitudinal incision was made along the left sternal border. The pectoralis major and minor muscles were separated layer by layer to expose the intercostal space. The operative space was then gently enlarged by penetrating the third-to-fourth intercostal muscles with curved hemostatic forceps, allowing the heart to exteriorize under pressure changes. In the MI and Col groups, the left anterior descending (LAD) coronary artery was permanently ligated with an 8-0 polypropylene suture at approximately 2 mm distal to the left auricle to standardize infarct location. Successful ligation was confirmed by blanching of the myocardium in the ligated region, accompanied by reduced regional wall motion. The heart was then immediately repositioned, and the muscle and skin layers were closed sequentially. Mice in the Sham group underwent the same anesthesia and thoracotomy procedures except that LAD ligation was not performed. During surgery, mice were maintained on a thermostatically controlled surgical table to preserve body temperature.

#### 2.3.3. Anti-Remodeling Drug Intervention

To mimic the clinical scenario in which patients receive delayed therapy after MI and to evaluate ECG as an effective indicator of post-MI remodeling, we adopted a delayed postoperative intervention design. On day 3 after MI surgery, a single intraperitoneal dose of colchicine (400 μg/kg) was administered to the Col group, whereas the Sham and MI groups received an equivalent dose of normal saline.

#### 2.3.4. Transparent Reporting of Animal Study Design

In this study, sample size was chosen with reference to previous MI mouse studies and the principle of reduction in animal use. Before surgery, animals were randomly assigned to the experimental groups. Owing to procedural constraints, strict allocation concealment was not implemented during surgery. Echocardiographic image acquisition and parameter measurements were performed by a trained cardiovascular surgeon, and only short-axis sectional images of the left ventricle with clear borders and complete contours were included for analysis. Echocardiographic evaluation, histological interpretation, and ECG interval measurements were all performed under blinded conditions. At the end point, surviving mice were euthanized by CO_2_ overdose before tissue collection.

### 2.4. Testing and Application of the FECMS

#### 2.4.1. Electrical Performance Testing of Flexible Serpentine Electrodes

Because the skin–electrode interface has both resistive and capacitive properties, impedance, rather than simple resistance, was used to characterize electrode performance. In ECG acquisition, interface impedance more directly reflects signal coupling quality and recording stability. Therefore, in this study, the electrical properties of the flexible serpentine electrodes were measured and characterized using an inductance–capacitance–resistance (LCR) meter (UTR2032, UNI-T, Guangdong, China). The intrinsic impedance and skin impedance of the flexible serpentine electrodes were measured by applying a 10 mV alternating current (AC) in the frequency range of 50 Hz to 10 kHz. To evaluate the stability of the electrodes during motion, impedance analysis was carried out under various stretching conditions.

#### 2.4.2. Accuracy Testing of FECMS

The ECG signals acquired with FECMS were compared with those recorded using a dedicated animal ECG acquisition system (BL-420N, TECHMAN, Chengdu, China) to verify the accuracy of ECG acquisition by the FECMS. We compared 10 paired recordings from the two systems, with one recording obtained from each of 10 mice, using mean squared error (MSE), mean absolute error (MAE), and signal-to-noise ratio (SNR) to quantify and evaluate signal quality and consistency.

#### 2.4.3. ECG Monitoring of Cardiac Remodeling

Building on FECMS, longitudinal ECG recordings were acquired from awake mice under the same standard Lead II configuration and used as electrophysiological readouts of post-MI remodeling. We used colchicine to generate treatment-associated differences in cardiac remodeling and recorded ECG parameter changes at multiple post-infarction time points (preoperative; postoperative days 1 and 3; post-treatment days 1, 3, 4, and 7; and postoperative day 14). Electrode placement and interval measurements were performed by a trained investigator blinded to both group allocation and recording time point. ECG signals were recorded for approximately 3–5 min at each time point. For each mouse, interval measurements were obtained by averaging 5–8 consecutive beats, and beats with obvious noise contamination were excluded from the analysis. As a supplementary sensitivity analysis, corrected QT (QTc) was additionally calculated using the Mitchell formula (QTc = QT/(RR/100)^1/2). Then, we compared ECG parameters across multiple time points and experimental groups, to test whether ECG can track the evolution of conduction and repolarization abnormalities during the remodeling process. ECG parameters were further combined with echocardiography, histological analysis and serum biomarkers of myocardial injury for correlation analysis, to verify the reliability of ECG in reflecting changes in cardiac function and structural remodeling.

### 2.5. Echocardiographic Validation of Functional Consistency

To validate the ability of ECG parameters to accurately track changes in cardiac function, echocardiography was performed on postoperative day 14 in all mice. Echocardiographic indices were then compared with ECG parameters acquired at the corresponding time point to assess the concordance of ECG with the functional reference standard. Cardiac function was assessed under isoflurane anesthesia using a high-resolution small-animal ultrasound system (Vevo 3100, FUJIFILM VisualSonics Inc., Toronto, ON, Canada). Ejection fraction (EF), fractional shortening (FS), left ventricular end-diastolic volume (LVEDV), and left ventricular end-systolic volume (LVESV) were calculated from three sequential cardiac cycles as the average values for each mouse.

### 2.6. Histological Validation of Structural Consistency

To evaluate myocardial fibrosis and inflammatory responses, and to assess whether ECG can reliably reflect structural cardiac remodeling, hearts were harvested on day 14 post-surgery and processed for fixation, paraffin embedding and histological sectioning.

#### 2.6.1. Masson Trichrome Staining

Paraffin-embedded heart sections were deparaffinized and rehydrated, followed by staining with Weigert’s iron haematoxylin for 5 min. Sections were then differentiated in acidic ethanol for 5 s and rinsed in water. Masson blue solution was applied for counterstaining and the slides were rinsed again, after which Ponceau S was used for 5 min. Sections were treated with phosphomolybdic acid for 1 min and subsequently stained with aniline blue for 1 min. Finally, the sections were differentiated, dehydrated, cleared and mounted with neutral gum.

#### 2.6.2. Haematoxylin–Eosin (HE) Staining

Paraffin-embedded heart sections were deparaffinized and rehydrated, then stained with haematoxylin for 3 min at 25 °C. After differentiation in 1% acid alcohol for 15 s, sections were blued in 0.6% ammonia water and rinsed in water. Slides were then stained with eosin for 2 min, washed in running water, dehydrated through graded alcohols, cleared, air-dried and mounted with neutral gum.

### 2.7. Molecular Biomarker Validation of Injury Consistency

To verify at the molecular level the reliability of ECG parameters in reflecting the recovery of myocardial injury, plasma troponin I (Tn-I) levels were measured in mice. On postoperative day 14, blood was collected by retro-orbital puncture under isoflurane anesthesia into anticoagulant tubes, kept on ice for 30 min and centrifuged at 3500 rpm for 10 min at 4 °C. The supernatant was then stored at −20 °C. Plasma Tn-I was quantified using a commercial enzyme-linked immunosorbent assay (ELISA) kit (Zhiyi Biotechnology Co. Ltd., Taizhou, China) according to the manufacturer’s instructions, with absorbance at 450 nm read on a microplate reader (Elx800, Bio-Tek Corporation, Winooski, VT, USA). Samples were assayed in triplicate and concentrations were calculated from a standard curve. Detailed information on the ELISA kit is provided in Supplementary Table S3.

### 2.8. Statistical Analysis

All statistical analyses were performed using SPSS 22.0 software. Data are presented as mean ± SD. Single-time-point group comparisons were analyzed by one-way ANOVA after testing homogeneity of variance, followed by Tukey’s HSD or Tamhane’s T2 post hoc tests as appropriate. Longitudinal ECG data were analyzed separately by repeated-measures ANOVA, and only animals completing the full follow-up were included in these analyses. Normality was assessed before analysis, and sphericity was evaluated using Mauchly’s test, with appropriate correction applied when necessary. Bonferroni adjustment was used for post hoc multiple comparisons. Correlation analyses were performed using two-tailed Spearman’s rank correlation. Statistical analyses were conducted using a 95% confidence interval, and a *p* value < 0.05 was considered statistically significant.

## 3. Results

### 3.1. Performance Characterization of the Flexible Serpentine Electrode

**Figure 2:**
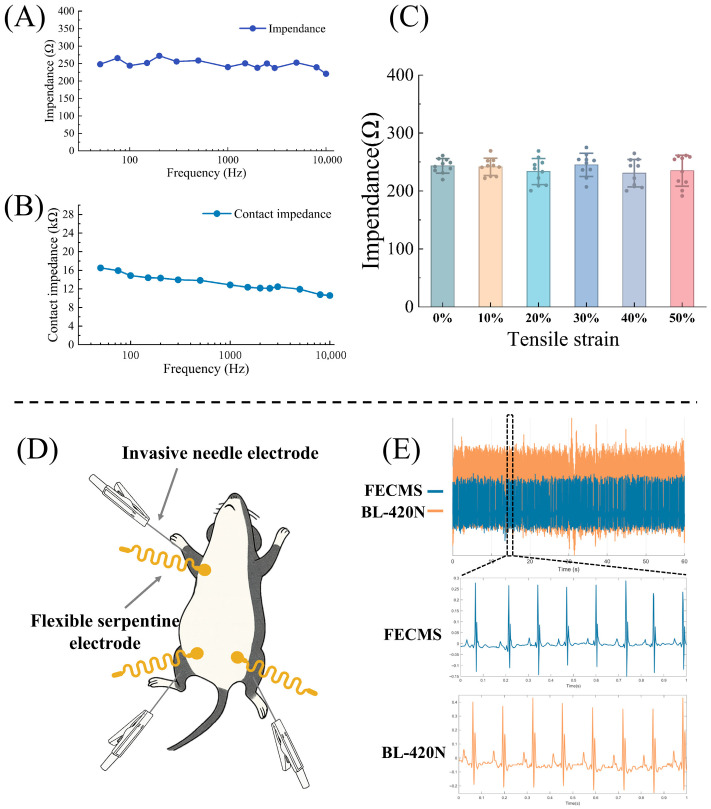
Performance characterization of the flexible serpentine electrode (*n* = 5) and comparison of ECG signal quality between FECMS and BL-420N. (**A**) Intrinsic impedance. (**B**) Electrode–skin interface impedance. (**C**) Impedance variation under different tensile strain conditions. (**D**) Schematic of electrode placement: invasive needle electrodes are connected to BL-420N, whereas non-invasive flexible serpentine electrodes are connected to FECMS. (**E**) Representative comparison of ECG waveforms recorded by the two systems, with the dashed box highlighting the magnified segment of the signal.

As shown in Figure 2A,B, the flexible serpentine electrodes maintain low intrinsic impedance and skin impedance across the 50 Hz–10 kHz range, demonstrating their suitability for stable surface ECG acquisition. As shown in Figure 2C, impedance of the flexible serpentine electrodes was measured under different stretching conditions that imitate animal movement or skin deformation, and only minor changes in impedance were observed, suggesting that the serpentine layout and flexible substrate effectively buffer the effect of mechanical deformation on electrical contact. These results suggest that the flexible serpentine electrode maintains stable impedance and signal fidelity under repeated tensile loading. Therefore, it is particularly suitable for long-term, continuous surface ECG monitoring in mice and provides a foundation for ECG-based treatment effects assessment in preclinical cardiac studies.

### 3.2. Accuracy of the FECMS

To verify the signal accuracy of the FECMS platform, we compared one-minute, two-lead ECG signals that were simultaneously recorded from the same mouse using FECMS and the BL-420N animal ECG system. Electrode positions are shown in Figure 2D. BL-420N recorded ECG using invasive needle electrodes, whereas FECMS used non-invasive flexible serpentine leads attached to the skin. As shown in Figure 2E, although the FECMS records ECG signals with lower amplitude, it captures waveforms comparable to those obtained with BL-420N. Minor discrepancies may be attributed to differences in electrode type, data preprocessing algorithms and hardware characteristics. Quantitative analysis showed low MSE and MAE and comparable SNR between the two systems, as presented in Table 1. The test results indicate that the FECMS retains the advantages of being non-invasive, flexible, lightweight and wireless, while offering a platform for long-term, low-interference ECG monitoring in mice.

### 3.3. Validation of ECG in Dynamic Monitoring of Cardiac Remodeling

**Figure 3:**
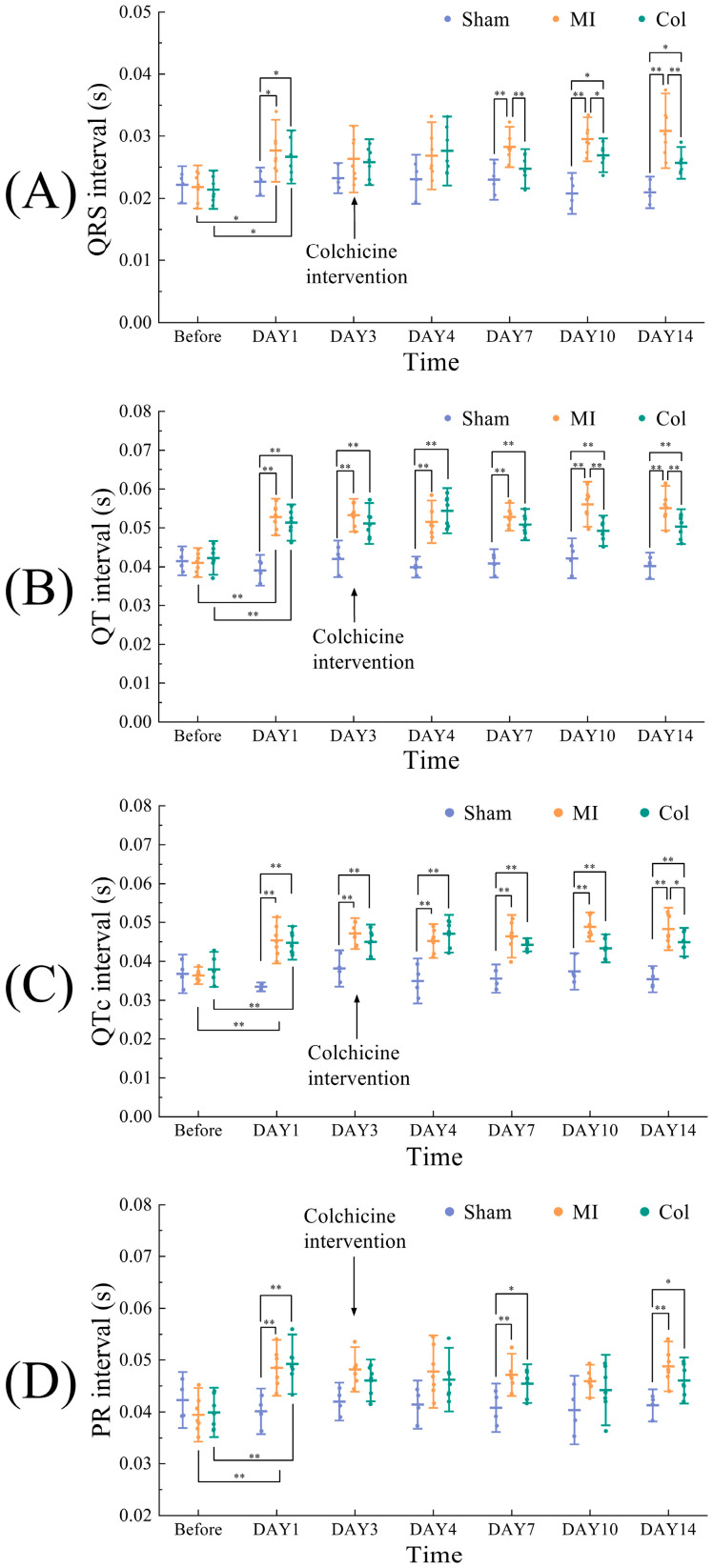
Temporal changes in selected ECG signal characteristics during post-MI cardiac remodeling. (**A**) QRS interval. (**B**) QT interval. (**C**) QTc interval. (**D**) PR interval. (* *p* < 0.05, ** *p* < 0.01).

To verify whether ECG parameters can track the time course of post-infarction cardiac remodeling and detect differences in remodeling severity, we compared ECG parameters in mice at multiple time points. The FECMS monitoring results are shown in Figure 3A–D. Before colchicine administration, ECG analysis after MI induction showed no significant differences in the major ECG parameters between the MI and Col groups, indirectly supporting comparable baseline electrophysiological injury status before treatment initiation and suggesting that the subsequent parameter differences were more likely related to remodeling divergence. Compared with the Sham group, MI mice displayed clear postoperative prolongation of the QRS, QT and PR intervals, indicating abnormal cardiac conduction and repolarization after MI. After colchicine treatment, ECG parameters in the Col group differed from those in the MI group. On day 3 after dosing, the QRS interval in the Col group was significantly shorter than that in the MI group (*p* < 0.05), indicating a reduction in the degree of ventricular conduction delay. On day 7 after dosing, both QRS and QT intervals in the Col group were significantly shorter than in the MI group (*p* < 0.05), closer to the levels of the Sham group, and this difference persisted until the end of the experiment, reflecting shortened conduction and repolarization delays. A supplementary QTc analysis showed the same overall directional trend as the raw QT analysis across groups and time points, further supporting the robustness of the observed QT changes in this model. The Col group exhibited a shorter mean PR interval than the MI group, suggesting that atrioventricular conduction disturbance was partly relieved after colchicine intervention. It can be clearly seen that anti-remodeling intervention improved postoperative ECG abnormalities after MI, as the previously markedly prolonged intervals did not further deteriorate but, instead, gradually shortened or stabilized over time, becoming significantly shorter than those in the MI group at multi-time-point and trending towards Sham levels.

By using the FECMS, we were able to continuously monitor the parallel time courses of remodeling improvement and ECG recovery under pharmacological intervention in the same animal, instead of limiting assessment to a single endpoint comparison.

### 3.4. Concordance of ECG with Cardiac Indices at Functional, Structural and Molecular Indicators

#### 3.4.1. ECG Concordance with Echocardiographic Indices

**Figure 4:**
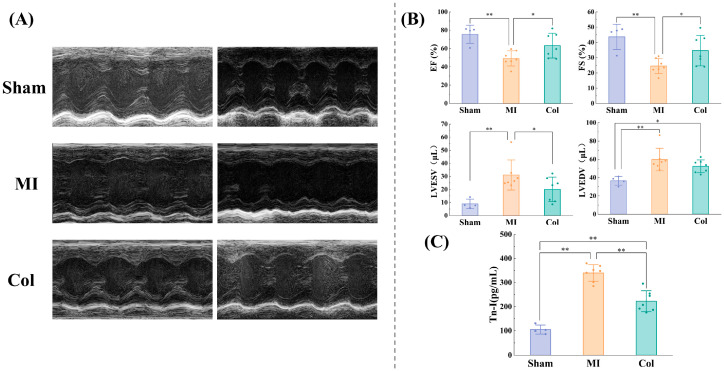
Echocardiographic indices and Tn-I levels 14 days after MI induction. (**A**) Representative transthoracic echocardiographic images from the Sham, MI and Col groups. (**B**) Quantitative comparison of key echocardiographic parameters (EF, FS, LVEDV and LVESV). (**C**) Tn-I levels in the Sham, MI and Col groups. (* *p* < 0.05, ** *p* < 0.01).

To determine whether changes in ECG parameters reflect alterations in cardiac mechanical function, we performed transthoracic echocardiography in all groups of mice 14 days after myocardial infarction. As shown in Figure 4A, short-axis views of the left ventricle were obtained to capture the full cardiac silhouette. Cardiac functional indices are shown in Figure 4B. Compared with the Sham group, the MI group had markedly reduced EF and FS together with significant increases in LVESV and LVEDV. These changes indicate the presence of left ventricular systolic dysfunction, chamber dilatation and prolongation of cardiac conduction pathways after MI. Compared with the MI group, the Col group exhibited significantly increased EF and FS together with a significantly reduced LVESV, with these indices approaching Sham values. In addition, LVEDV in the Col group showed a modest decrease compared with the MI group. These findings suggest that colchicine treatment substantially improved left ventricular systolic function and partially constrained left ventricular chamber dilatation, thereby making the ventricular state closer to normal physiological levels. Taken together with the preceding ECG monitoring results, improvements in cardiac functional indices after anti-remodeling intervention were directionally aligned with ECG changes, indicating that recovery of cardiac function and attenuation of electrophysiological abnormalities occurred in parallel.

#### 3.4.2. ECG Concordance with Histopathological Findings

To determine whether changes in ECG parameters reflect myocardial histological changes, we performed Masson’s trichrome and HE staining on hearts from each group, examining fibrotic remodeling and inflammatory responses as structural correlates of the ECG findings.

**Figure 5:**
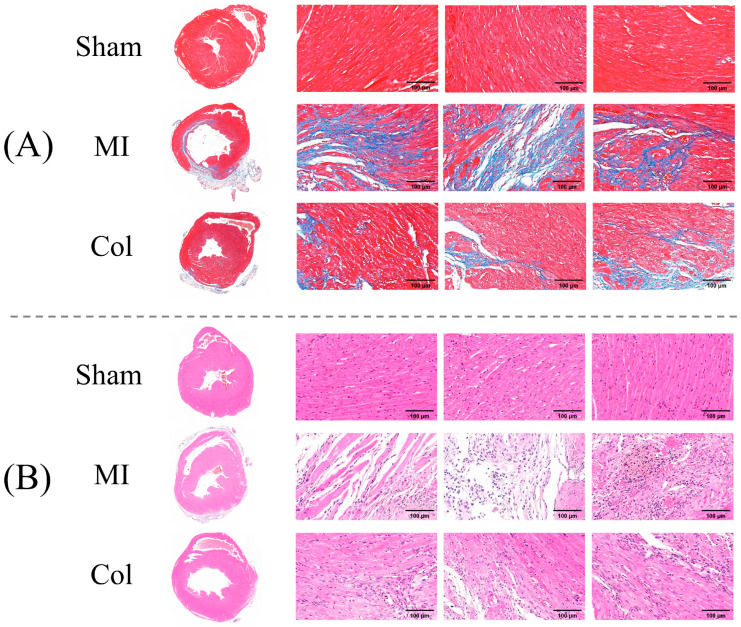
Histological evaluation of mouse hearts. (**A**) Representative Masson-trichrome-stained sections of myocardium. (**B**) Representative HE-stained sections.

In Masson-trichrome-stained sections of myocardium, aniline blue stains collagen fibers blue and ponceau/acid fuchsin stains cytoplasm and myofibers red. The Masson-trichrome-stained results are shown in Figure 5A. In the Sham group, myocardial architecture appeared orderly arranged with virtually no fibrosis or myofiber disruption. In contrast, the MI group displayed dense blue-stained collagen within the infarct and border zones, partial myocardial fiber rupture and a markedly higher degree of fibrosis than in the Sham group. Compared with the MI group, the Col group displayed markedly reduced collagen deposition and a lower fibrotic burden, while myocardial structural continuity was preserved. Consistent with these histological changes, ECG monitoring revealed significantly shorter QRS and QT intervals in mice receiving anti-remodeling treatment than in the MI group. This finding suggests that, under anti-remodeling treatment, reduced fibrotic burden was accompanied by partial alleviation of ventricular conduction delay and repolarization abnormalities.

In HE staining sections of myocardium, haematoxylin stains nuclei dark purple and eosin stains cytoplasm and extracellular matrix pink. The HE staining results are shown in Figure 5B. In the Sham group, myocardial cells showed normal morphology and orderly alignment, without evident inflammatory infiltration. By contrast, the MI group exhibited marked inflammatory cell infiltration, disorganized myocyte architecture and enlarged, hyperchromatic nuclei. Compared with the MI group, the Col group showed clearly reduced inflammatory infiltration, a more orderly arrangement of myocardial cells and relatively intact intercellular connections, indicating a reduction in local inflammatory burden and tissue damage. The corresponding ECG monitoring results showed that the PR interval in the Col group tended to be more stable than in the MI group, suggesting a partial alleviation of atrioventricular conduction abnormalities.

Therefore, histological improvements after anti-remodeling treatment were directionally consistent with ECG improvements, providing representative morphological support for an association between electrophysiological abnormalities and tissue-level fibrosis and inflammation in this model.

#### 3.4.3. ECG Concordance with Molecular Markers

To evaluate whether ECG parameter changes reflect the extent of myocardial injury, we measured plasma Tn-I levels in each group of mice using ELISA. The results are shown in Figure 4C. Compared with the Sham group, the MI group exhibited markedly elevated plasma Tn-I levels, consistent with pronounced cardiomyocyte necrosis after MI. In contrast, the Col group had significantly lower Tn-I levels than the MI group, suggesting that colchicine reduced the extent of myocardial injury and allowed more viable cardiomyocytes to participate in conduction and repolarization. Taken together with the preceding ECG monitoring results, the decrease in plasma Tn-I levels after anti-remodeling treatment was aligned with the direction of QRS and QT interval changes, providing endpoint molecular support for the association between ECG parameters and myocardial injury severity.

#### 3.4.4. Correlation of ECG with Cardiac Indices at Functional, Structural and Molecular Indicators

**Figure 6:**
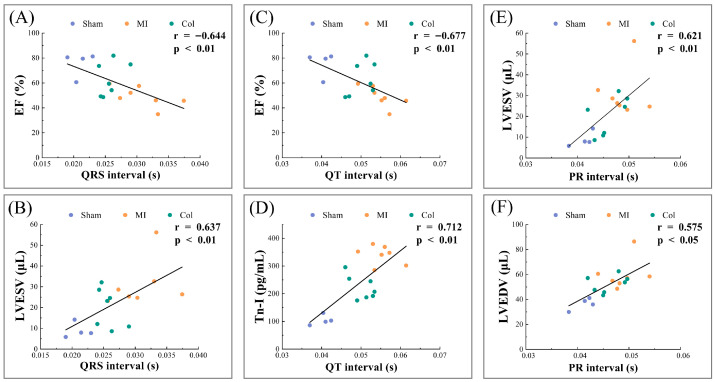
Spearman correlations between endpoint ECG intervals and functional, structural and molecular cardiac indices. (**A**) QRS interval versus EF. (**B**) QRS interval versus LVESV. (**C**) QT interval versus EF. (**D**) QT interval versus Tn-I. (**E**) PR interval versus LVESV. (**F**) PR interval versus LVEDV.

To examine how ECG parameters relate to indices of cardiac function, structure and injury, we performed Spearman correlation analyses. At the functional level, we analyzed correlations between ECG parameters and cardiac functional indices. As shown in Figure 6A–C, the QRS interval was inversely related to EF (r = −0.644, *p* < 0.01) and positively related to LVESV (r = 0.637, *p* < 0.01), and the QT interval, likewise, exhibited an inverse correlation with EF (r = −0.677, *p* < 0.01). It can be considered that, after anti-remodeling treatment, improvements in ECG parameters were directionally aligned with improvements in cardiac function, suggesting an association between electrophysiological changes and functional recovery in this model. At the injury level, we analyzed correlations between ECG parameters and molecular markers of myocardial injury. As shown in Figure 6D, QT interval duration was positively associated with Tn-I concentration (r = 0.712, *p* < 0.01), indicating that more severe myocardial injury is associated with more prolonged repolarization. Combined with the ELISA results, these findings suggest that attenuation of myocardial injury and stabilization of repolarization evolve in parallel after anti-remodeling treatment, indicating that ECG parameters may capture the relationship between relief of myocardial injury and drug efficacy. At the structural level, we analyzed correlations between ECG parameters and structural remodeling indices. As shown in Figure 6E,F, PR interval duration was positively associated with LVESV (r = 0.621, *p* < 0.01) and LVEDV (r = 0.575, *p* < 0.05). Combined with the histological staining in the Col group showing less fibrosis, reduced inflammatory infiltration and attenuated chamber dilatation, these results suggest that improvements in ECG parameters are associated with attenuation of cardiac remodeling, which provides preliminary evidence that ECG parameters may reflect structural remodeling-related changes in this model.

## 4. Discussion

With the development of cardiovascular drugs, monitoring and quantifying treatment effects in animal models are essential to bridge basic research and clinical translation. Yet conventional evaluation methods rarely permit continuous tracking of drug-induced dynamic changes in cardiac structure and function within the same animal. In this context, complementary longitudinal physiological readouts may be valuable for tracking remodeling and treatment response over time.

The FECMS developed in this study is a flexible and lightweight implementation for repeated, low-burden ECG acquisition in mice, supporting multi-time-point recordings and improving the practicality of longitudinal ECG interval assessment in small-animal studies. Compared with conventional wet or rigid electrodes, the flexible serpentine electrodes designed in this study may offer practical advantages in conformability and recording burden for repeated longitudinal ECG monitoring.

The monitoring results showed that MI mice exhibited persistent prolongation of the QRS, QT and PR intervals, whereas anti-remodeling-treated mice exhibited shorter ECG intervals than untreated MI mice, with values approaching those of the Sham group at multiple time points. These interval changes paralleled treatment-associated improvements in functional, structural, and injury-related measures, suggesting that ECG intervals obtained from FECMS may provide complementary longitudinal readouts of remodeling progression in this pharmacological post-MI setting. These observations are consistent with previous evidence emphasizing the prognostic and remodeling-related value of ECG intervals in cardiovascular disease, providing a rationale for further investigating ECG as an electrophysiological marker of post-MI remodeling in preclinical studies.

At the functional level, prior studies suggest that adverse ventricular remodeling may alter effective conduction pathways, which could contribute to QRS prolongation [20]. In the colchicine-treated mice, the combination of improved systolic performance and reduced chamber dilatation was directionally consistent with shorter QRS intervals, which may reflect partial normalization of ventricular conduction. This interpretation is consistent with the observation that QRS intervals in the Col group were closer to Sham levels than those in the MI group. At the structural level, fibrotic scar formation after MI is known to disrupt myocardial conduction pathways and increase local tissue resistivity, which may contribute to prolongation of the QRS and QT intervals [21,22]. Inflammatory burden may also contribute to atrioventricular conduction abnormalities, which could be reflected by PR interval changes [23]. Anti-remodeling treatment reduced inflammatory infiltration and fibrotic burden, which may have preserved the continuity of myocardial and atrioventricular conduction tissue, consistent with the ECG improvements observed in the Col group. At the injury level, myocardial damage reduces the amount of viable myocardium available for electrical conduction and repolarization, which may contribute to QRS and QT prolongation [24,25]. Under anti-remodeling treatment, attenuation of myocardial injury may be associated with better preservation of excitable myocardium. Meanwhile, the lower myocardial injury burden observed in the Col group was directionally consistent with shorter QRS and QT intervals, indirectly supporting an association between reduced injury burden and improved electrophysiological status. Together, these multi-level associations support the interpretation that ECG interval changes may provide complementary information on functional, structural, and injury-related changes of post-infarction remodeling under pharmacological intervention.

Here, we suggest that future evaluation of cardiovascular therapies should also consider longitudinal trajectories of ECG parameters across the entire follow-up period as an additional dimension of assessment. Stabilization or partial declines in these parameters may reflect suppression of adverse cardiac remodeling or mitigation of myocardial injury. When individuals exhibit larger-magnitude, more persistent interval prolongation, especially if this is accompanied by abnormalities across multiple ECG indices, they may reflect more severe remodeling or injury and should be regarded as potential high-risk phenotypes.

Prior studies suggest that initiating therapy on day 3 after MI is associated with better outcomes [26]. In addition, the 0.4 mg/kg dose range has precedent in murine myocardial injury studies, where a single intraperitoneal dose of 400 μg/kg colchicine was used, whereas higher doses (≥1 mg/kg) showed early toxicity [27]. Previous studies have also suggested that overly early or excessive suppression of early inflammation may impair tissue repair and increase the risk of left ventricular rupture [28,29,30]. Therefore, we adopted a delayed single-dose intervention design, with ECG monitoring covering the interval from the inflammatory peak to fibrotic remodeling. Although values did not fully return to Sham levels, the results support the proof-of-concept evidence of ECG measurements to track the temporal evolution of post-MI remodeling under non-acute treatment conditions. However, we acknowledge that the translational relevance of a single delayed dose may differ from the continuous or multi-dose regimens typically employed in clinical anti-inflammatory therapy.

This study has several limitations. First, validation was confined to an MI mouse model with a relatively small sample size, and future studies with larger cohorts will be needed to provide a more precise assessment. In particular, the study examined only a single drug and dosing condition, and the follow-up period was limited to 14 days, so more extended post-MI remodeling was not assessed. In addition, broader engineering validation and subsequent clinical validation of the FECMS remain needed.

Despite these drawbacks, the flexible wearable monitoring device developed in this study demonstrates considerable promise as a platform for cardiac function monitoring. In the future, with the assistance of clinicians, this system could be leveraged for drug screening, efficacy evaluation and investigation of drug-related adverse effects, while providing a foundation for physiological signal monitoring in animal models.

## 5. Conclusions

In summary, we developed the FECMS as a flexible, non-invasive system for repeated ECG acquisition in mice and provided proof-of-concept evidence that longitudinal ECG interval changes can be used to track treatment-associated remodeling in a colchicine-treated post-MI model. Its applicability was evaluated in a myocardial infarction model subjected to anti-remodeling pharmacological intervention. This long-term monitoring capability may facilitate the study of dynamic ECG changes associated with post-MI remodeling progression. Thus, this study supports the feasibility of using longitudinal ECG interval changes as complementary readouts alongside established endpoint-based assessment methods in this pharmacological post-MI setting.

## Figures and Tables

**Table 1:** Signal quality and error analysis results of ECG signals collected by BL-420N and FECMS (*n* = 10).

Metric	Mean ± SD
MSE	0.005080513 ± 0.001647
MAE	0.04719051 ± 0.009187
SNR of BL-420N	22.8853 ± 4.101201
SNR of FECMS	24.4749 ± 3.020621

## Data Availability

All data that support the findings of this study are included within the article.

## Supplementary Materials

The following supporting information can be downloaded at: https://www.mdpi.com/article/10.3390/s26092814/s1, Table S1. Comparison of representative ECG monitoring approaches; Table S2. Summary of animal numbers; Table S3. ELISA kit information.
