# Supplementary material for: A Flexible Wearable Electronics System for Electrocardiographic Assessment of Colchicine Therapy for Post-MI Remodeling
Source: Sensors (Basel). 2026 Apr 30;26(9):2814. doi: 10.3390/s26092814 (PMC13165562; doi:10.3390/s26092814)
Supplement: Supplementary file 1 [file sensors-26-02814-s001.zip › sensors-4274752-supplementary.pdf]

## Supplementary Materials

**Table S1.** Comparison of representative ECG monitoring approaches.

| Approach | Invasiveness | Flexibility or wearability | Wireless capability | Longitudinal suitability |
|---|---|---|---|---|
| Implantable telemetric ECG (ETA-F10 telemetry device) [11] | High (abdominal implantation with subcutaneous chest leads) | Low | Yes | High |
| Implantable telemetry systems (DSI HDS11/PAC10/C50-PXT and Stellar PBTA-M-C) [15] | High (surgical implantation of device and ECG leads) | Low | Yes | High |
| Rigid bed-integrated surface ECG system (AD8232 module integrated with the easyPET.3D mouse bed) [17] | Low (paw surface contact with dry electrodes under anesthesia) | Low | No | Moderate |
| Rigid plate-based surface ECG system (sensor array with 15 gold plated electrodes) [18] | Low(paw contact with dry electrode plate) | Low | No | Moderate |
| Integrated monitoring systems (stainless-steel microneedle electrodes and AD8233) [19] | Moderate(microneedles puncture the stratum skin surface) | High | Yes | Moderate |
| FECMS | Low(body surface flexible electrodes) | High | Yes | High |

**Table S2**. Summary of animal numbers.

| Group | Baseline (allocated) | Post-surgery survivors | End-point survivors |
|---|---|---|---|
| **Sham** | **4** | **4** | **4** |
| **MI** | **8** | **7** | **7** |
| **Col** | **8** | **7** | **7** |

**Table S3**. ELISA kit information.

| Item | Description |
|---|---|
| **Standard concentrations** | **0, 75, 150, 300, 600, 1200 pg/mL （0.3ml*6tubes）** |
| **Standard curve range** | **0–1200 pg/mL** |
| **Sample Diluent** | **6.0ml** |
| **HRP-Conjugate reagent** | **10.0ml** |
| **20X Wash solution** | **25ml** |
| **Chromogen Solution A** | **6.0ml** |
| **Chromogen Solution B** | **6.0ml** |
| **Stop Solution** | **6.0ml** |
| **Curve fitting method** | **Linear regression** |
